# Identification and Characterization of BmNPV m6A Sites and Their Possible Roles During Viral Infection

**DOI:** 10.3389/fimmu.2022.869313

**Published:** 2022-03-16

**Authors:** Xing Zhang, Yaxin Zhang, Jun Pan, Chengliang Gong, Xiaolong Hu

**Affiliations:** ^1^ School of Biology & Basic Medical Science, Soochow University, Suzhou, China; ^2^ Agricultural Biotechnology Research Institute, Agricultural Biotechnology, and Ecological Research Institute, Soochow University, Suzhou, China

**Keywords:** BmNPV, m6A modification, *ie-1*, BmYTHDF3, viral replication

## Abstract

*Bombyx mori* nucleopolyhedrovirus (BmNPV) is one of the most serious pathogens and causes serious economic losses in sericulture. At present, there is no epigenetic modification of BmNPV transcripts, especially of m6A, and this modification mediates diverse cellular and viral functions. This study showed that m6A modifications are widespread in BmNPV transcripts in virally infected cells and the identified m6A peaks with a conserved RRACH sequence. m6A sites predominantly appear in the coding sequences (CDS) and the 3′-end of CDS. About 37% of viral genes with m6A sites deleted from the viral genome did not produce any infectious virions in KOV-transfected cells. Among the viral genes related to replication and proliferation, *ie-1* mRNA was identified with a higher m6A level than other viral genes. The m6A sites in the *ie-1* mRNA may be negatively related to the protein expression. Viral replication was markedly inhibited in cells overexpressed with BmYTHDF3 in a dose-dependent manner, and a contrary effect was found in si-BmYTHDF3-transfected cells. Collectively, the identification of putative m6A modification in BmNPV transcripts provides a foundation for comprehensively understanding the viral infection, replication, and pathobiology in silkworms.

## Introduction

*Bombyx mori* nucleopolyhedrovirus (BmNPV) is one of the most serious pathogens and causes serious economic losses in sericulture (1). Its genome is circular double-strand DNA with 130 kb and encodes 141 putative proteins (2). BmNPV is a classical member of baculoviruses with two types of virions in the whole life cycle. The budded virus (BV) is responsible for the horizontal transmission among the cells, and the occlusion-derived virus (ODV) is responsible for the vertical transmission (3). BmNPV belongs to the nuclear-replicated virus and is inevitably modified by a series of host modification enzymes, including m6A modification.

N^6^-Methyladenosine (m6A) is one of eukaryotic mRNA’s most abundant internal modifications. It is also a dynamic reversible RNA modification, which plays an important role in the structure, positioning, and function of RNA (4, 5). m6A is involved in various biological processes, including stress response, fertility, stem cell differentiation, circadian rhythm, microRNA biogenesis, and cancer occurrence development (6–9). m6A modification systems in eukaryotic cells mainly include methyltransferase (Writer), demethylases (Eraser), and m6A reader protein (Reader) (10). The motif domain of RRAmCH (R=G/A, G > A, H=A, C, U) is recognized by Writer and Reader in the cells (5). m6A modification on mRNA is catalyzed by the methyltransferase complex (Writers), including methyltransferase-like 3/14 (METTL3/METTL14) and other cofactors (11). Demethylase fat mass and obesity-associated protein (FTO) and alkB homologous protein (ALKBH) as Erasers are mainly responsible for the demethylation of m6A modification (12). The YTH domain-containing m6A reader proteins (YTHDF1, YTHDF2, YTHDF3, YTHDC1, and YTHDC2) and the homogeneous nuclear protein family (HNRNPA2B1 and HNRNPC) can recognize the m6A site or region by participating in the regulation of mRNA stability, mRNA structure, mRNA nuclear export, and mRNA splicing and translation (13–19). To date, m6A modifications appear in many DNA and RNA viral mRNAs *via* the host m6A modification machinery (20). m6A modifications were reported to play important roles in the viral life cycle. Both influenza A virus (IAV) mRNA and viral genome RNA are m6A modified, and the expression level of a viral gene can be upregulated by cis-regulation of this m6A modification (21). m6A modification appears in the RNA genome of hepatitis C virus (HCV), and simultaneous silencing of m6A methyltransferase-like 3/14 (METTL3/METTL14) significantly increased the expression level of the viral NS5A protein. By silencing the demethylase FTO gene, the non-structural 5A (NS5A) protein level was significantly reduced (22). Similarly, the effects of Writer and Eraser on Zika virus (ZIKV) were consistent with HCV (23). The m6A modification system inhibits HCV production by affecting viral assembly, while inhibition of ZIKV is through its effects on viral replication (23–25). After vesicular stomatitis virus (VSV) infection, the level of DEAD-box 46 (DDX46) RNA helicase and demethylase ALKBH5 in the nucleus increased significantly, which inhibited antiviral protein expression and the host innate immune response to VSV (26). When the human immunodeficiency virus (HIV) infects lymphocytes, the level of m6A methylation is elevated in both viral RNAs and host cellular RNAs. Downregulation of METTL3 and METTL14 gene expression levels in CD4 T lymphocytes inhibits HIV replication, while downregulation of ALKBH5 gene expression levels promotes HIV replication (25). METTL3 also interacted with enterovirus 71 (EV71) RNA-dependent RNA polymerase (RdRp) 3D. The 3D protein stability and transcriptional efficiency were increased by increasing 3D sumoylation/ubiquitination modification to promote viral replication (27). The m6A modification of human metapneumovirus (HMPV) can help the virus successfully evade host immune system recognition, thus promoting HMPV replication and gene expression (28). It has been reported that mRNAs of DNA viruses are modified by m6A and participate in the viral life cycle. It was found that Kaposi sarcoma-associated herpesvirus (KSHV) replication was inhibited by knockdown of the METTL3 or YTHDF1-3 gene (29). m6A modification has a positive regulatory effect in the life cycle of Simian virus 40 (SV40), and overexpression of YTHDF2 can accelerate virus replication (30). The m6A modification site located in the 3′UTRs and 3′ epsilon loop of the hepatitis B virus (HBV) genome is not conducive to the stability of HBV RNA. The m6A modification site located in the 5′ ϵ ring of the HBV genome has a positive regulatory effect on pregenomic RNA (pgRNA) reverse transcription (31). EBV virus (EBV) transcripts are many m6A modification sites. YTHDF1 can recognize m6A sites accelerating viral RNA uncapping and recruit RNA degradation complexes to inhibit viral infection and replication (32). m6A modification in adenovirus (ADV) transcripts involves regulating splicing and viral RNA expression (33). The porcine epidemic diarrhea virus (PEDV) genome contains multiple m6A modification sites and negatively regulates the virus infection (34). Our preliminary findings showed that the expression levels of writers (BmMETTL3 and BmMETTL3) and reader (BmYTHDF3) of m6A machinery in BmN cells were negative to the expression of viral structural protein VP39 in BmNPV infection (35). These studies indicate that both DNA and RNA viruses are regulated by the m6A modification system in host cells during viral infection, but the m6A modification system has different regulatory mechanisms for different viruses.

BmNPV belongs to the nuclear-replicated virus. Whether m6A modification is introduced into the viral transcripts in the viral proliferation process, and whether this apparent modification is beneficial for the virus to evade the detection of the host’s innate immune system, is still unclear. In this study, the m6A modification was investigated by methylated RNA immunoprecipitation (MeRIP) sequencing from the BmNPV-infected midgut, and m6A sites were widespread in the viral transcripts. Furthermore, the m6A machinery negatively mediated the viral replication. The results will uncover the roles of BmNPV transcript m6A modification in the viral life cycle and provide a scientific base for anti-BmNPV.

## Materials and Methods

### Sample Preparation

The domesticated silkworm strain (Jingsong×Haoyue) was reared in our lab with fresh mulberry leaves at 25°C. The polyhedrin (1*10^6^) of BmNPV stored in our lab at Soochow University was evenly coated on the mulberry leaves with 4*4 cm for the first day of 5 instar larvae. 48 h postinfection, the midguts were collected and extracted for total RNA. BmN cells were cultured in TC-100 medium with 10% fetal bovine serum (HyClone, Logan, UT, USA) in a 26°C incubator. The recombinant BmNPV-GFP was gifted by Professor Xiaofeng Wu from the Zhejiang University.

### Methylated RNA Immunoprecipitation Sequencing

30 silkworm larvae were infected by BmNPV, and the midguts were used to extract the total RNA with the TRIzol reagent (Invitrogen, Carlsbad, CA, USA). The total RNA was fragmented with the corresponding buffer (10 mM ZnCl_2_, 10 mM Tris–HCl pH 7.0). The obtained fragments were captured with the m6A antibody, and the corresponding libraries were utilized for MeRIP sequencing (MeRIP-Seq), and further bioinformatics analysis was also conducted by Shanghai OE Biotech. Co., Ltd. (Shanghai, China). The sequencing data were deposited in the NCBI with accession numbers (SRR10141250, SRR10141249, SRR10141248, and SRR10141247) (35). BmNPV T3 (accession number: L33180.1) was used as the reference genome for mapping.

### MeRIP-qPCR

The method for preparation of total RNA for MeRIP-qPCR was the same protocol as MeRIP sequencing, except that total RNA was not treated with the fragmentation buffer. Briefly, BmNPV-infected BmN cells were lysed with lysis buffer. The lysed cells were separated into parts. One part of lysate was incubated with an anti-m6A antibody, and another part was incubated with IgG control (Rabbit) conjugated to Protein A/G beads in 1 ml RNA immunoprecipitation (RIP) buffer. After incubation at 4°C overnight, the beads were washed with wash buffer 3 times, and the RIP samples were reverse transcribed into cDNA using the PrimeScript RT Reagent Kit (TaKaRa, Dalian, China) and subjected to RT-qPCR. Translation initiation factor 4a (TIF-4A) was used as a housekeeping gene (36). The specific primers for each viral gene used in real-time PCR are listed in **Supplementary Table 1**. All experiments were conducted three times.

### Plasmids and siRNA

The genome loci of *ie-1* in BmNPV T3 (L33180.1) are 116994.118748 and were synthesized by the Sangon Biotechnology company (Shanghai, China). The DNA sequence was inserted into the Kpn I and Xba I sites in the pIZT-V5/his vector (Invitrogen, Frederick, MD, USA) for pIZT-V5his-ie. A mutant fragment of *ie-1* was mutated A to T at the potential m6A sites. This mutant DNA sequence was also synthesized by the Sangon Biotechnology company (Shanghai, China) and was inserted into the sites of Kpn I and Xba I in the pIZT-V5/his vector for pIZT-V5his-ie-1mut. pIZT-BmYTHDF3 and siRNA (si-BmYTHDF3) specifically targeting BmYTHDF3 were reported in our previous study (35).

### Western Blotting

BmN cells (1*10^5^) were transfected with 2 μg pIZT-V5/his vector and pIZT-V5his-ie-1mut with Lipofectamine (Roche, Mannheim, Germany), respectively. 48 h post-transfection, the cells were harvested and lysed in RIPA lysis buffer (Beyotime, Shanghai, China) supplemented with protease inhibitors (Beyotime, Shanghai, China). The expression levels of IE-1 and enhanced green fluorescence protein (EGFP) were detected with anti-His, anti-tubulin, and anti-EGFP antibodies (Proteintech, Rosemont, IL, USA) as the primary antibodies, and horseradish peroxidase-conjugated goat anti-mouse IgG (Proteintech, USA) was used as the secondary antibody. The reactions were visualized using an ECL reagent (Sangon, Shanghai).

### Overexpression and Depletion of BmYTHDF3 in BmN Cells and BmNPV Infection

BmN cells (1*10^5^ cells/well) were transfected with a recombinant expression plasmid (pIZT-BmYTHDF3) with the transfection reagent. 48 h post-transfection, BmN cells were infected with BmNPV-GFP (MOI = 2) and harvested for extraction of total proteins at 12 h postinfection for Western blotting. Meanwhile, BmN cells (1*10^5^ cells/well) were transfected with specific siRNA targeting BmYTHDF3 (si-BmYTHDF3) with the transfection reagent. 48 h post-transfection, BmN cells were infected with BmNPV-GFP (MOI = 2) and harvested for extraction of total proteins at 12 h postinfection for Western blotting. The expression of the *Bm59* gene of BmNPV was used as the indicator of viral replication (37).

### Bioinformatics

m6A modification sites on the interested RNA sequences were carried out by the SRAMP prediction server (38). The motif discovery from the sets of sequences was investigated by HOMER software (39). The flexibility of protein local structures was studied through (i) the B-factor of the X-ray experiment and (ii) the fluctuation of residues during molecular dynamics simulations (40). The Flexibility Prediction was utilized to analyze the flexibility classes (0, 1, and 2 represent rigid, intermediate, and flexible, respectively) according to the observed dynamic properties from the target protein sequences.

## Results

### m6A Modifications Are Widespread in BmNPV Transcripts in Virally Infected Cells

**Figure 1:**
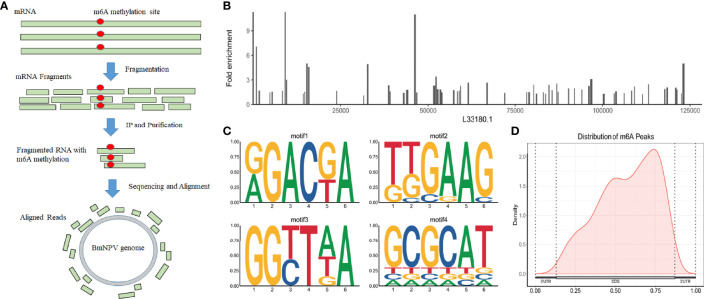
BmNPV transcripts are m6A modified. **(A)** The workflow of MeRIP is for the detection of m6A modification in BmNPV transcripts. **(B)** The distribution of m6A peaks on the genome of BmNPV T3 (L33180.1). **(C)** The conserveness of the RRACH motifs within the identified m6A peaks using HOMER. **(D)** Distribution of m6A peaks on viral transcripts including 5′-UTR, CDS, and 3′-UTR.

BmNPV-infected silkworm midguts were collected and sequenced with methylated RNA immunoprecipitation combined with high-throughput sequencing (MeRIP-Seq) to understand the m6A modification in mRNAs, including host mRNAs and viral mRNAs (**Figure 1A**). Our previous study found that thousands of m6A peaks were identified from BmNPV-infected tissues, and the host m6A modification system was related to the expression of the viral gene. However, this modification is still a puzzle whether it appears in the BmNPV mRNAs. To better understand the post-transcription modification, especially m6A sites on the viral genes and the potential roles in the viral life cycle, we compared the MeRIP-Seq data with the BmNPV T3 (L33180.1) reference genome and found that 74 regions with high potential m6A methylation (m6A peak) were identified in 59 mRNA transcripts of BmNPV (**Figure 1B** and **Table 1**). Among these m6A peaks, the mean size of the m6A peak is 262 bp, while the maximum and minimum peaks are 599 and 135 bp, respectively. HOMER motif enrichment analysis was utilized to analyze the RRACH motif conservation in 74 identified m6A peaks. The results showed that four conserved RRACH motifs were identified from the alignments between the *de novo* motif and known motifs and met the requirement of the motif domain of RRAmCH (R=G/A, G > A; H=A, C, U) (**Figure 1C**). The positions of m6A sites were analyzed to understand the appearance of m6A sites in viral mRNAs. The results indicated that m6A modification mainly appeared in coding sequences (CDS) and the 3′-end of CDS (**Figure 1D**). These results suggested a potential biofunctional significance for m6A modification in viral mRNA.

**Table 1:** The information of m6A peaks on BmNPV transcripts from the MeRIP sequencing.

Chromosome/genome	ChromStart	ChromEnd	Name	Score	Strand	Sizes
L33180.1	57541	57675	Orf_61	0.022	–	134
L33180.1	91856	91999	Orf_95	0.0018	–	143
L33180.1	85439	85584	he65	6.60E-05	–	145
L33180.1	78238	78383	bro-c	0.00025	+	145
L33180.1	82971	83117	Orf_87	5.60E-07	–	146
L33180.1	9016	9163	Orf_9	0.00095	+	147
L33180.1	71601	71748	Orf_76	0.11	+	147
L33180.1	127894	128041	lef-2	0.00018	+	147
L33180.1	72394	72394	odv-e25	0.49	+	148
L33180.1	122464	122612	pe38	1.30E-66	+	148
L33180.1	11024	11172	arif-1	0.0036	–	148
L33180.1	25369	25517	39k	0.0098	–	148
L33180.1	26006	26154	39k	0.97	–	148
L33180.1	121197	121345	ie-2	2.20E-07	–	148
L33180.1	121636	121784	ie-2	2.20E-07	–	148
L33180.1	14226	14375	Orf_14	0.00052	+	149
L33180.1	30886	31035	gta	0.033	+	149
L33180.1	52237	52386	Orf_54	0.037	+	149
L33180.1	83635	83784	vp80	0.018	+	149
L33180.1	93966	94115	Orf_97	1.00E-05	+	149
L33180.1	80205	80355	lef-5	7.90E-10	+	150
L33180.1	36040	36190	lef-8	1.80E-05	–	150
L33180.1	125383	125533	bro-d	0.00071	–	150
L33180.1	108994	109144	p74	7.90E-06	–	150
L33180.1	106072	106223	p35	0.00098	+	151
L33180.1	4703	4854	Orf_5	0.0035	–	151
L33180.1	61422	61610	Orf_67	0.0017	–	188
L33180.1	56767	56957	Orf_60	2.90E-05	–	190
L33180.1	16947	17139	dbp	0.001	–	192
L33180.1	108182	108376	p26	2.10E-05	+	194
L33180.1	53267	53461	lef-3	0.004	–	194
L33180.1	53797	53991	lef-3	0.01	–	194
L33180.1	78756	78950	38k	0.0021	–	194
L33180.1	19447	19643	Orf_21	6.60E-05	–	196
L33180.1	31475	31672	gta	0.0012	+	197
L33180.1	71903	72100	odv-e25	0.0093	+	197
L33180.1	90186	90383	Orf_94	0.0043	–	197
L33180.1	8263	8461	bv/odv-e26	0.0021	+	198
L33180.1	23755	23955	fgf	6.30E-18	+	200
L33180.1	86801	87002	Orf_90	6.30E-19	+	201
L33180.1	111302	111545	me53	0.00025	–	243
L33180.1	9435	9679	Orf_10	0.0034	–	244
L33180.1	112889	113136	ie-0	0.03	+	247
L33180.1	866	1113	orf1629	2.00E-05	–	247
L33180.1	46527	46774	gp37	0.00019	–	247
L33180.1	5301	5550	Orf_5	0.003	–	249
L33180.1	70170	70449	Orf_74	3.20E-07	–	279
L33180.1	17751	18045	iap1	0.00055	+	294
L33180.1	84525	84823	vp80	0.0017	+	298
L33180.1	80936	81234	Orf_85	0.00048	–	298
L33180.1	87150	87451	Orf_90	6.30E-19	+	301
L33180.1	15729	16053	pkip	0.00013	–	324
L33180.1	38589	38925	Orf_40	2.00E-96	+	336
L33180.1	32541	32881	Orf_36	0.0021	+	340
L33180.1	115589	115929	odv-ec27	0.002	+	340
L33180.1	120710	121052	ie-2	2.20E-07	–	342
L33180.1	101807	102150	p24	0.0017	+	343
L33180.1	42816	43191	Orf_47	0.021	–	375
L33180.1	57051	57432	Orf_60	2.90E-05	–	381
L33180.1	118211	118602	ie-1	0.0024	+	391
L33180.1	52483	52879	Orf_54	1.10E-05	+	396
L33180.1	58288	58685	vlf-1	0.0018	–	397
L33180.1	45981	46402	Orf_51	6.30E-08	+	421
L33180.1	117432	117871	ie-1	0.0029	+	439
L33180.1	1458	1903	orf1629	1.40E-05	–	445
L33180.1	43802	44248	fp	0.0019	–	446
L33180.1	103057	103507	pp34	0.00022	+	450
L33180.1	103556	104030	Orf_109	0.01	+	474
L33180.1	66807	67281	p30	4.00E-04	–	474
L33180.1	122855	123345	pe38	1.30E-66	+	490
L33180.1	51448	51942	Orf_54	1.10E-05	+	494
L33180.1	95815	96354	Orf_101	9.10E-08	+	539
L33180.1	96473	97059	lef-7	2.50E-17	–	586
L33180.1	100092	100690	gp64/67	4.20E-05	–	598

### Viral Genes With m6A Sites Were Classified Into Four Phenotypes

The KOV (bacmid)s were subdivided into four phenotypes (A to D) according to the results from the knockout BmNPVs (KOVs) for each viral gene using the lambda red recombination system (41). The defined phenotypes of type-A and -B KOVs could produce infectious viruses, but types C and D did not produce any infectious virions in KOV-transfected cells (2). According to the appearance of m6A in the viral genes, we found 33, 4, 17, and 5 viral genes in types A, B, C, and D, respectively (**Table 2**).

**Table 2:** The KOV (bacmid)s were subdivided into four phenotypes (A to D) according to the results from the knockout BmNPVs (KOVs) for each viral gene using the lambda red recombination system.

A	B	C	D
pe38	bro-d	lef-2	lef-5
arif-1	me53	odv-e25	lef-8
39k	ie-0	vp80	lef-3
ie-2	pkip	38k	ie-1
gta		gp64/67	orf1629
he65		odv-ec27	
bro-c		vlf-1	
p74		dbp	
p35		p45	
p26		bv/odv-c42	
bv/odv-e26		dnaJ domain	
fgf		Orf_61	
p30		Orf_76	
gp37		Orf_54	
iap1		Orf_67	
fp		Orf_90	
p24		Orf_109	
pp34			
pif-3			
pif-1			
chaB-like			
lef-7			
Orf_9			
Orf_14			
Orf_5			
Orf_60			
Orf_21			
Orf_94			
Orf_10			
Orf_74			
Orf_36			
Orf_51			
Orf_101			

A and -B KOVs could produce infectious viruses, but type-C and -D did not produce any infectious virions in KOV-transfected cells.

### Validation of m6A Modification in Viral Genes

**Figure 2:**
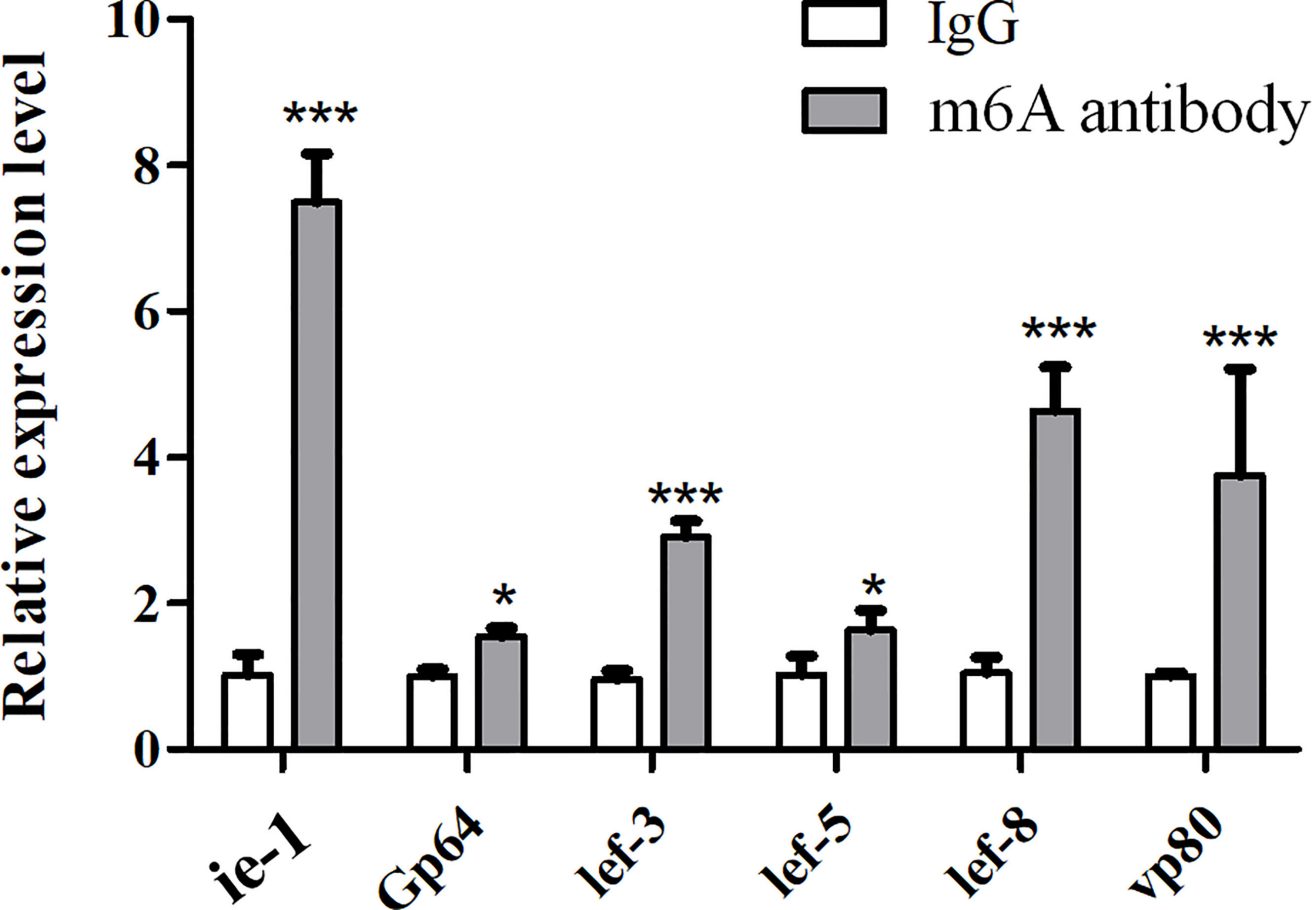
Validation of m6A modification in viral replication-related genes using m6A antibody immunoprecipitation combined with qPCR (MeRIP-qPCR). The IgG or anti-m6A antibody enrichment of the viral mRNAs was measured by MeRIP-qPCR in BmNPV-infected BmN cells. *P < 0.05 and ***P < 0.001.

To know the existence of the m6A site in viral genes, multiple genes related to viral replication (*lef-8*, *lef-5*, *lef-3*, *VP80*, *GP64/78*, and *ie-1*) were selected for validation using m6A antibody immunoprecipitation combined with qPCR (MeRIP-qPCR). The results showed that all these viral genes could be enriched by the m6A antibody and detected by qPCR (real-time PCR) using the corresponding primers of each gene (**Supplementary Table 1**). Compared to the relative expression levels of viral genes in the complex of the m6A antibody and viral mRNAs with m6A modification, the results showed that *ie-1* mRNA with a higher m6A level than other viral genes was identified from the complex (**Figure 2**), indicating that enriched m6A modification in the *ie-1* gene may be related to the transcription or translation.

### The Expression Level of IE-1 Was Related to the m6A Sites

**Figure 3:**
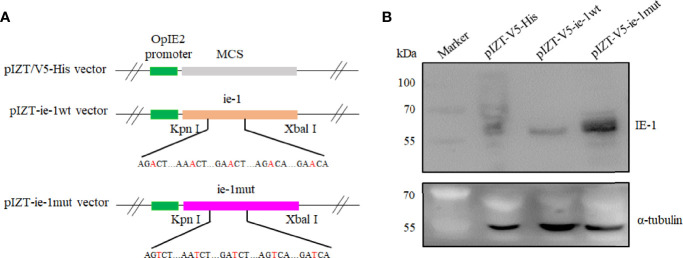
Construction of the ie-1wt and ie-1mut plasmids and detection of the expression level of IE-1 in BmN cells transfected with plasmids. **(A)** The wild-type and mutant ie-1 plasmids were constructed by cloning a 1,752-nt length fragment (remove the TAA) encompassing the m6A peak region in the *ie-1* mRNA and a mutant fragment with A > T mutations at the potential m6A sites, respectively. **(B)** IE-1 expression was detected from the plasmid-transfected BmN cells with Western blotting assay. pIZT-V5-His plasmid as the control.

**Figure 4:**
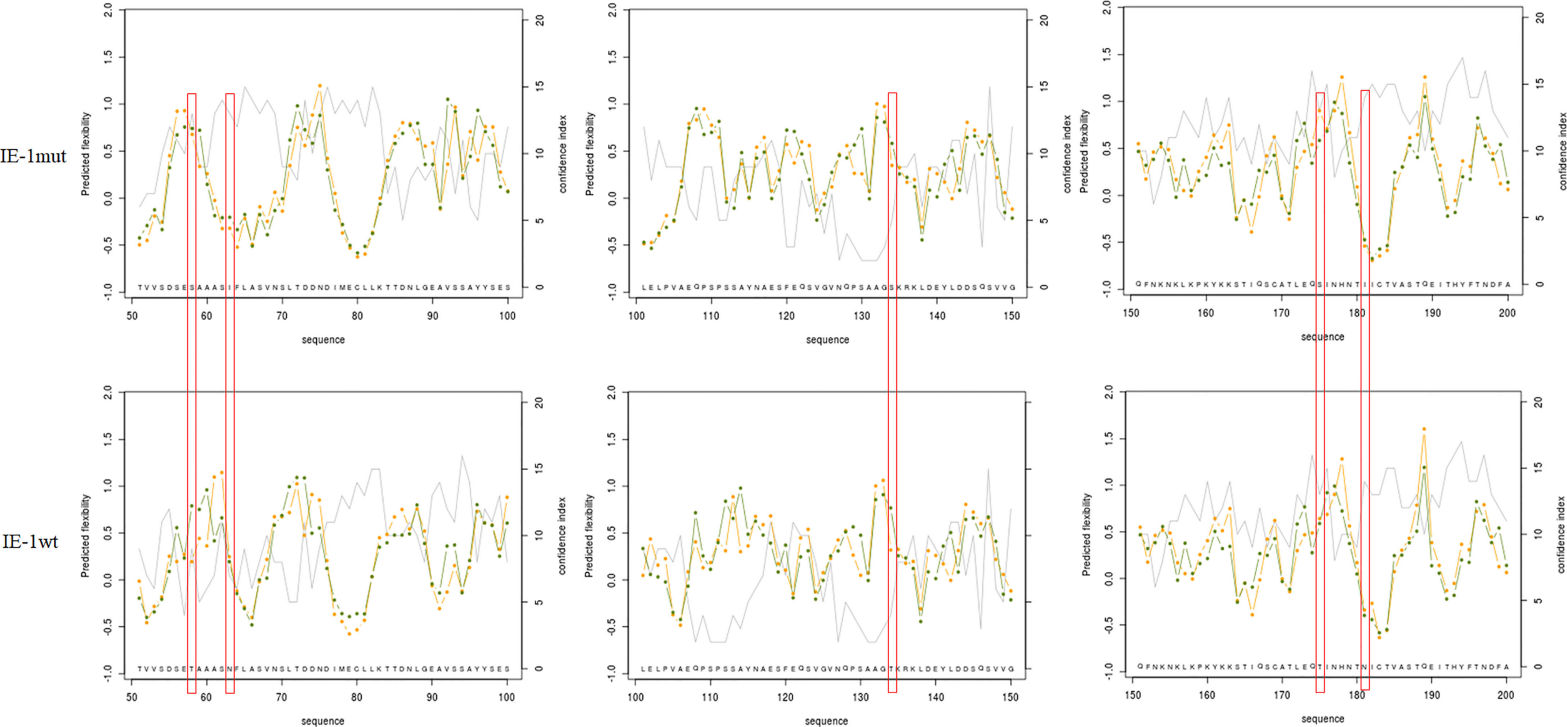
The flexibility of protein local structures was studied through (i) the B-factor of the X-ray experiment and (ii) the fluctuation of residues during molecular dynamic simulations. IE-1wt (58T, 63N, 134T, 175T, and 181N) > IE-1mut (58S, 63I, 134S, 175S, and 181I).

To further validate the function of m6A sites in the *ie-1* mRNA, the wild-type and mutant ie-1 plasmids were constructed by cloning a wild-type 1,752-nucleotide (nt) length fragment (remove the TAA) encompassing the m6A peak region in the *ie-1* mRNA and a mutant fragment with A > T mutations at the potential m6A sites, respectively (**Figure 3A**). The plasmids were transfected into BmN cells for Western blotting assay, which exhibited a higher IE-1 expression level on the mutant plasmid than wild-type ie-1 (**Figure 3B**), confirming that the presence of m6A sites in the *ie-1* mRNA may be related to its expression. To exclude the influence of codon on protein expression, the flexibility of protein local structures was studied through (i) the B-factor of the X-ray experiment and (ii) the fluctuation of residues during molecular dynamics simulations (40). The prediction results showed that these 5 amino acid mutants in the IE-1 had not altered the protein flexibility (**Figure 4**).

### m6A Reader (BmYTHDF3) Negatively Regulated the Viral Replication

**Figure 5:**
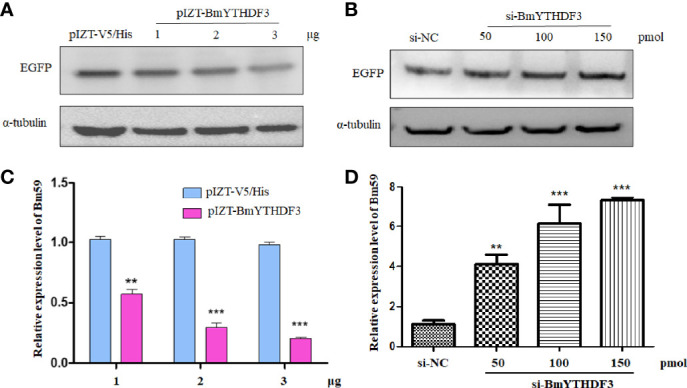
Knockdown of BmYTHDF3 suppressed BmNPV infection and replication. **(A)** The infection of BmNPV in BmN cells transfected with pIZT-BmYTHDF3 (1, 2, and 3 μg) or pIZT-V5/His as control following BmNPV-GFP infection was detected by Western blotting. **(B)** The infection of BmNPV in BmN cells transfected with si-BmYTHDF3 (50, 100, and 150 pmol) or si-NC as control following BmNPV-GFP infection was detected by Western blotting. **(C)** The relative expression level of *Bm59* in BmN cells transfected with pIZT-BmYTHDF3 (1, 2, and 3 μg) or pIZT-V5/His as control following BmNPV-GFP infection was detected by real-time PCR. **(D)** The relative expression level of *Bm59* in BmN cells transfected with si-BmYTHDF3 (50, 100, and 150 pmol) or si-NC as control following BmNPV-GFP infection was detected by real-time PCR. **p < 0.05 and ***p < 0.001.

Our previous study concluded that cytoplasmic YTH-domain family 3 (BmYTHDF3) was indicated as the m6A reader of the m6A machinery in silkworm, *Bombyx mori*, and the m6A modification system was predicted to play important roles in the BmNPV infection (35). The appearance of m6A modification in the *ie-1* gene means that BmYTHDF3 could recognize these m6A sites. However, the exact roles of BmYTHDF3 in the replication of BmNPV are still not clear, and we used the recombinant expression plasmid (pIZT-BmYTHDF3) and specific siRNA targeting BmYTHDF3 transfected into BmN cells followed by BmNPV-GFP infection for examining the roles of BmYTHDF3 in the viral replication. The Western blotting assay showed that the EGFP was markedly inhibited in cells overexpressed with BmYTHDF3 in a dose-dependent manner, and a contrary effect was found in si-BmYTHDF3-transfected cells (**Figures 5A, B**). Meanwhile, a real-time PCR assay obtained similar results *via* detecting the *Bm59* gene expression level in BmN cells overexpressed with BmYTHDF3 and transfected with si-BmYTHDF3 (**Figures 5C, D**), suggesting that BmYTHDF3 could inhibit the BmNPV infection in BmN cells *via* recognizing the m6A sites in the viral gene.

## Discussion

Increasing data indicated that m6A modification not only exists in the cellular mRNAs but also is widespread in the viral transcripts, including DNA and RNA viruses. Viral m6A modifications have been reported as pro-viral or antiviral functions depending on the virus species, cell type, and viral RNA location (20). We showed that m6A modification is widespread in BmNPV transcripts in virally infected cells. Viral genes with m6A sites were classified into four phenotypes according to the results from the knockout BmNPVs (KOVs) for each viral gene using the lambda red recombination system (41). We demonstrated that ie-1 mRNA with a higher m6A level than other viral genes was examined, and the expression level of IE-1 was related to the m6A sites in the viral ie-1 gene. Furthermore, m6A reader protein BmYTHDF3 suppressed the BmNPV infection in BmN cells, maybe recognizing the m6A sites in the viral gene.

m6A machinery regulates cellular RNAs of viral RNAs by writers, erasers, and readers in cells (42). The appearance of m6A modification in viral RNAs and cellular m6A modification systems played vital roles in the viral life cycle. Our previous study found that the cellular mRNAs with m6A modification were altered following BmNPV infection and the expression levels of writers and readers negatively mediated BmNPV infection (35). However, the precise regulation of m6A modification on viral infection was vague. Therefore, we made assumptions about widespread m6A sites in the BmNPV transcripts like other viruses, and the viral RNA with m6A sites could be regulated by cellular m6A reader proteins. Presently, any reports showed that BmNPV transcripts could be modified by the m6A machinery. MeRIP-Seq data were aligned with the BmNPV T3 (L33180.1) reference genome, and 74 regions with high potential m6A methylation (m6A peak) were distributed in 59 viral mRNA transcripts. Among these m6A peaks, four conserved RRACH motifs were identified as other species. The enrichment analysis of m6A peaks on viral mRNAs showed that most were distributed on the CDS and 3′-CDS of viral mRNAs. It is known that m6A modification was found near the 3′ UTR and stop codon in mammals and mice (43). The methylation landscape across human adult tissues showed m6A sites preferentially around stop codons, essential for maintaining basic cellular function. Most of the m6A in the genes with tissue specificity were found in the 5′-UTR (44), suggesting that these viral genes with m6A sites may be essential for distinct regulatory effects in the aspect of host range, viral replication, assembly, and host–virus interaction. 22/59 viral genes were demonstrated to be required to produce infectious viruses, and the m6A site appearance in these genes may be related to the viral replication. Furthermore, several viral genes related to viral replication were selected to be validated by MeRIP-qPCR, and the results also showed that m6A modifications were enriched in these selected viral genes. Among them, the modification level of m6A on the ie-1 gene was higher than other genes, indicating that these m6A sites might regulate their transcription or translation.

In our previous study, we found that the expression level of VP39 was decreased in BmN cells overexpressed with BmMETTL3 or BmYTHDF3, while the reverse results were obtained in the BmN cell depletion of BmMETTL3 or BmYTHDF3 using corresponding specific siRNAs (35). These suggested that the m6A modification system plays an important role in BmNPV infection with an unknown mechanism. The role of the YTHDF readers on viral replication was extensively examined by depletion and overexpression studies in numerous DNA and RNA viruses (29, 32, 45). Increasing data showed that YTHDF readers regulate the mRNA fate of cellular or viral m6A RNAs. It is described to regulate splicing, nuclear export, cap-independent translation, and decay (46). To better understand the regulatory effects of m6A machinery on the expression of viral genes or viral replication, the expression level of IE-1 was detected from BmN cells transfected with the wild-type and mutant *ie-1* plasmids, and Western blotting assay exhibited a higher IE-1 expression level on the mutant plasmid than wild-type *ie-1*, confirming that the presence of m6A sites in the *ie-1* mRNA may be related to its expression. When these mutant sites were introduced into *ie-1*, any adverse effect on protein flexibility was detected. Using the BmNPV-GFP-infected BmN cells overexpressed with BmYTHDF3 and transfected with si-YTHDF3, we found that the EGFP and viral gene *Bm59* were significantly decreased and increased, respectively. These results suggest that BmYTHDF3 suppressed the BmNPV infection in BmN cells *via* recognizing the m6A sites in viral genes. The m6A modification system has different regulatory mechanisms for different viruses (45, 47). The appearance of m6A modification on viral RNA will be used as a molecular marker for evading host innate immune recognition of non-self RNA (28). Altogether, many BmNPV transcripts have been modified by the m6A machinery, including many genes related to viral infectivity. BmYTHDF3 suppressed the BmNPV infection by recognizing the m6A sites in viral genes, resulting in impairment of viral gene expression, avoiding viral overproliferation.

## Data Availability Statement

The datasets presented in this study can be found in online repositories. The names of the repository/repositories and accession number(s) can be found in the article/**Supplementary Material**.

## Author Contributions

XH and CG contributed to the conception and design of the study. XZ, YZ, and JP performed the detection and analysis. XZ and XH wrote the first draft of the manuscript. CG wrote sections of the manuscript. All authors contributed to the manuscript revision and read and approved the submitted version.

## Funding

This study was supported by the National Key R&D Program of China (2019YFA0905200), the China Postdoctoral Science Foundation (2019M651952), and the Priority Academic Program of Development of Jiangsu Higher Education Institutions.

## Conflict of Interest

The authors declare that the research was conducted without any commercial or financial relationships that could be construed as a potential conflict of interest.

## Publisher’s Note

All claims expressed in this article are solely those of the authors and do not necessarily represent those of their affiliated organizations, or those of the publisher, the editors and the reviewers. Any product that may be evaluated in this article, or claim that may be made by its manufacturer, is not guaranteed or endorsed by the publisher.
