# Supplementary material for: Identification and Characterization of BmNPV m6A Sites and Their Possible Roles During Viral Infection
Source: Front Immunol. 2022 Mar 16;13:869313. doi: 10.3389/fimmu.2022.869313 (PMC8966388; doi:10.3389/fimmu.2022.869313)
Supplement: Supplementary file 1 [file Table_1.doc]

| Gene name | Forward primers (5’-3’) | Reverse primers (5’-3’) |
| --- | --- | --- |
| reTIF-4A | GAATGGACCCTGGGACACTT | CTGACTGGGCTTGAGCGATA |
| ie-1 | CGACTACAATTCCAACAGGT | ATTTCAAACGGCTTTACTTC |
| Gp64 | ACGTGATTATTGGCTACAAGG | GCAAATCTTCTTTGCCCACAG |
| Lef-3 | GTCTGACGACATCAATGAGGT | GAGTTGACCACGTTCCAATTC |
| Lef-5 | CGTTCAACTTTTCGTCTACCG | GTGTTGTTGAACAGCTTTGCC |
| Lef-8 | GCTATTCAAGAACGCAAGTC | ATTGCTTGTCTAAGCGCGTG |
| Vp80 | GAACATTCAGGGTTACGATAG | AATCTTTCCGCTTCCTGTTC |
| Bm59 | GACCAATATCTAAACAGCGAC | TTCGTCAAACACCATGGCGTT |

Supplementary Table 1 The primers were used for real-time PCR assay.
